# Real-time intra-fraction motion management in breast cancer radiotherapy: analysis of 2028 treatment sessions

**DOI:** 10.1186/s13014-018-1072-4

**Published:** 2018-07-16

**Authors:** D. Reitz, G. Carl, S. Schönecker, M. Pazos, P. Freislederer, M. Niyazi, U. Ganswindt, F. Alongi, M. Reiner, C. Belka, S. Corradini

**Affiliations:** 1 Department of Radiation Oncology, University Hospital, Marchioninistr 15, 81377 Munich LMU, Munich, Germany; 2 Department of Radiation Oncology, Medical University, Innsbruck, Austria; 3 Department of Radiation Oncology, Sacro Cuore Don Calabria Hospital, Verona, Negrar Italy; 4 University of Brescia, Brescia, Italy

**Keywords:** Radiotherapy, Intrafraction motion, Breast cancer, Optical surface scanner

## Abstract

**Background:**

Intra-fraction motion represents a crucial issue in the era of precise radiotherapy in several settings, including breast irradiation. To date, only few data exist on real-time measured intra-fraction motion in breast cancer patients. Continuous surface imaging using visible light offers the capability to monitor patient movements in three-dimensional space without any additional radiation exposure. The aim of the present study was to quantify the uncertainties of possible intra-fractional motion during breast radiotherapy.

**Material and methods:**

One hundred and four consecutive patients that underwent postoperative radiotherapy following breast conserving surgery or mastectomy were prospectively evaluated during 2028 treatment sessions. During each treatment session the patients’ motion was continuously measured using the Catalyst™ optical surface scanner (C-RAD AB, Sweden) and compared to a reference scan acquired at the beginning of each session. The Catalyst system works through an optical surface imaging with light emitting diode (LED) light and reprojection captured by a charge coupled device (CCD) camera, which provide target position control during treatment delivery with a motion detection accuracy of 0.5 mm. For 3D surface reconstruction, the system uses a non-rigid body algorithm to calculate the distance between the surface and the isocentre and using the principle of optical triangulation. Three-dimensional deviations and relative position differences during the whole treatment fraction were calculated by the system and analyzed statistically.

**Results:**

Overall, the maximum magnitude of the deviation vector showed a mean change of 1.93 mm ± 1.14 mm (standard deviation [SD]) (95%-confidence interval: [0.48–4.65] mm) and a median change of 1.63 mm during dose application (beam-on time only). Along the lateral and longitudinal axis changes were quite similar (0.18 mm ± 1.06 mm vs. 0.17 mm ± 1.32 mm), on the vertical axis the mean change was 0.68 mm ± 1.53 mm. The mean treatment session time was 154 ± 53 (SD) seconds and the mean beam-on time only was 55 ± 16 s. According to Friedman’s test differences in the distributions of the three possible directions (lateral, longitudinal and vertical) were significant (*p* < 0.01), in post-hoc analysis there were no similarities between any two of the three directions.

**Conclusion:**

The optical surface imaging system is an accurate and easy tool for real-time motion management in breast cancer radiotherapy. Intra-fraction motion was reported within five millimeters in all directions. Thus, intra-fraction motion in our series of 2028 treatment sessions seems to be of minor clinical relevance in postoperative radiotherapy of breast cancer.

## Background

Over the last decade, modern radiotherapy (RT) techniques including intensity modulated radiation therapy (IMRT), volumetric-modulated arc therapy (VMAT) or hypofractionated radiotherapy regimens have been introduced in breast cancer irradiation. These technological and technical improvements in RT require an accurate and reliable patient positioning. [1] Inter-fractional variabilities in patient positioning are typically handled by X-ray images acquired with electronic portal imaging devices (EPID) before a treatment session additional to laser-assisted positioning. As a consequence patients are exposed to additional ionizing radiation. [2–4]

Patient movements during breast cancer radiotherapy and especially during dose delivery have been limited in scope in clinical context, although it might have an impact on adequate planning target volume (PTV) setup margins.[5, 6] Nowadays, optical surface imaging offers the possibility to monitor patient movements in real-time using a non-invasive approach without any additional radiation exposure. The system offers a concrete option for safe patient positioning and has been analyzed by several study groups. [7–9] Crop et al. showed that the optical system Catalyst™ was superior to laser-assisted positioning. In addition, Stieler et al. reported a reduction in cone beam computed tomography (CBCT) scans needed in patients with a fixed tumour to the surface relationship.[10, 11] In regard to intra-fractional motion, Ricotti et al. recently reported results from breast cancer RT using an infrared light system with surface skin markers. The mean baseline drift was 0 ± 0.7 mm in right-left, − 0.5 ± 1.7 mm in inferior-superior and − 1.4 ± 1.8 mm in posterior-anterior direction. [12] Furthermore, Gaisberger et al. used 3D body surface imaging and noticed intrafractional shifts of 1.2 ± 0.7 mm during the first 2 min of observation. [13]

The aim of the present study was to quantify the uncertainties of possible intra-fractional chest motion during breast radiotherapy by using the real-time optical surface imaging system Catalyst™.

## Methods

Between October, 2016 and June, 2017, 104 women diagnosed with breast cancer, who received postoperative RT at the Department of Radiation Oncology, University Hospital, LMU Munich were consecutively recruited in a prospective study. RT-related information, tumour and patient characteristics of each patient were retrieved from medical records. The conventional fractionated RT scheme to the breast or chest wall was 2Gy to a total dose of 50Gy in 25 fractions, a sequential boost to the tumour bed was applied with a dose of 5× 2Gy or 8× 2Gy. Hypofractionated treatments were applied with a dose of 2.67Gy per fraction to a total dose of 40Gy. Patients who received RT in deep inspiration breath hold (DIBH) were excluded from the present analysis.[14, 15] The study was approved by the local ethics committee of the University Hospital, LMU Munich (No. 352–16 ex 09/2016) and registered at German Clinical Trials Register (DRKS-ID: DRKS00011407). Written informed consent was obtained for all patients.

For each patient a planning CT scan using a Toshiba Aquillion LB CT Scanner (Toshiba Medical Systems Corporation, Japan) was carried out and target delineation and treatment planning were performed. The clinical target volume (CTV_breast/chestwall_) encompassed the chest wall or the glandular breast parenchyma. The planning target volume (PTV_breast/chestwall_) was obtained by adding a 5 mm margin to the CTV_breast/chestwall_. In cases where a boost was applied, CTV_boost_ included the tumor bed, visible surgical clips and anatomical distortion. The PTV_boost_ was generated using a 5 mm isotropic expansion on CTV_boost_. Patient set-up was in a supine position on a positioning device (WingSTEP™, IT-V, Austria) with both arms elevated above the head. 3D conformal radiation therapy was used. Treatment planning was performed using the Oncentra 4.5.2 software (Elekta AB, Stockholm, Sweden). All plans consisted of two opposing tangential beams for the breast/chest wall with the addition of some subfields to increase dose homogeneity, as well as anterior/posterior fields for regional nodal irradiation (RNI). RNI included lymph node levels IV, III, Rotter lymph nodes and some parts of lymph node level II according to the ESTRO-guidelines. [16]

Before the first treatment fraction, patients were positioned using the skin marks and a laser-alignment-system followed by the acquisition of iView™ portal images (Elekta AB, Sweden) to verify proper positioning. Furthermore, a surface reference image of the region of interest was acquired using the Catalyst system after the patient had been finally positioned.

### Catalyst system

**Fig. 1:**
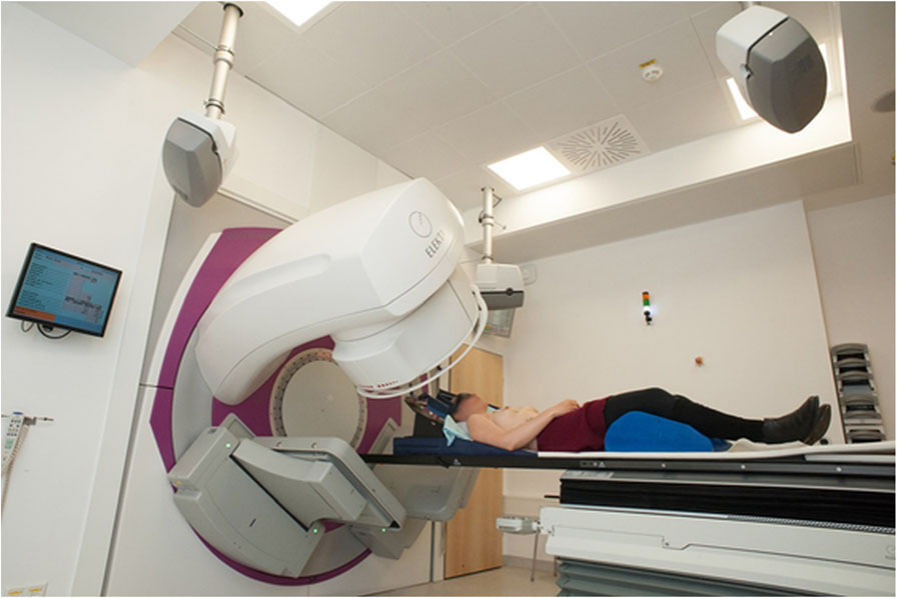
Image of the installed Catalyst™ system (showing the three Catalyst cameras) at the Department of Radiation Oncology, University Hospital, LMU Munich

The Catalyst HD optical surface scanner manufactured by C-RAD (Uppsala, Sweden) uses visible light to scan the body surface using three cameras placed in 120-degree angles relative to each other. Figure 1 shows the three Catalyst cameras beside the linear accelerator (LINAC). The Catalyst system works through optical surface imaging with LED light (blue: λ = 450 nm) and reprojection captured by a CCD camera (green: λ = 528 nm; red: λ = 624 nm), which provides target position control during treatment delivery. By the usage of three cameras, measurements are performed based on optical triangulation and a three-dimensional surface image of the body is calculated and compared to an initial reference image. The software calculates the relative displacement vectors in all three dimensions by using a non-rigid body algorithm. [17]

The Catalyst camera system and the C-RAD Catalyst-software tool c4D were utilized for real time observation of patients’ movements during every single RT session. The system compares the real-time image with a reference image and calculates absolute position deviations from the surface-projected isocentre. The system’s frame rate is approximately 200 frames per second and the c4D-software calculates and outputs a mean value over several samples.

According to the Catalyst system manual (2016 C-RAD Positioning AB) the system has a motion detection accuracy within 0,5 mm for a rigid body when the couch is in a fixed position during treatment delivery, which held always true in the present setting (3D plans only). These data were verified using phantom measurements in our department. As long as the view of the target was not restricted by any camera, a translational accuracy of 1.17 ± 0.3 mm in the lateral and longitudinal directions and 0.5 ± 0.2 mm in the vertical direction were measured.

### Data processing

**Fig. 2:**
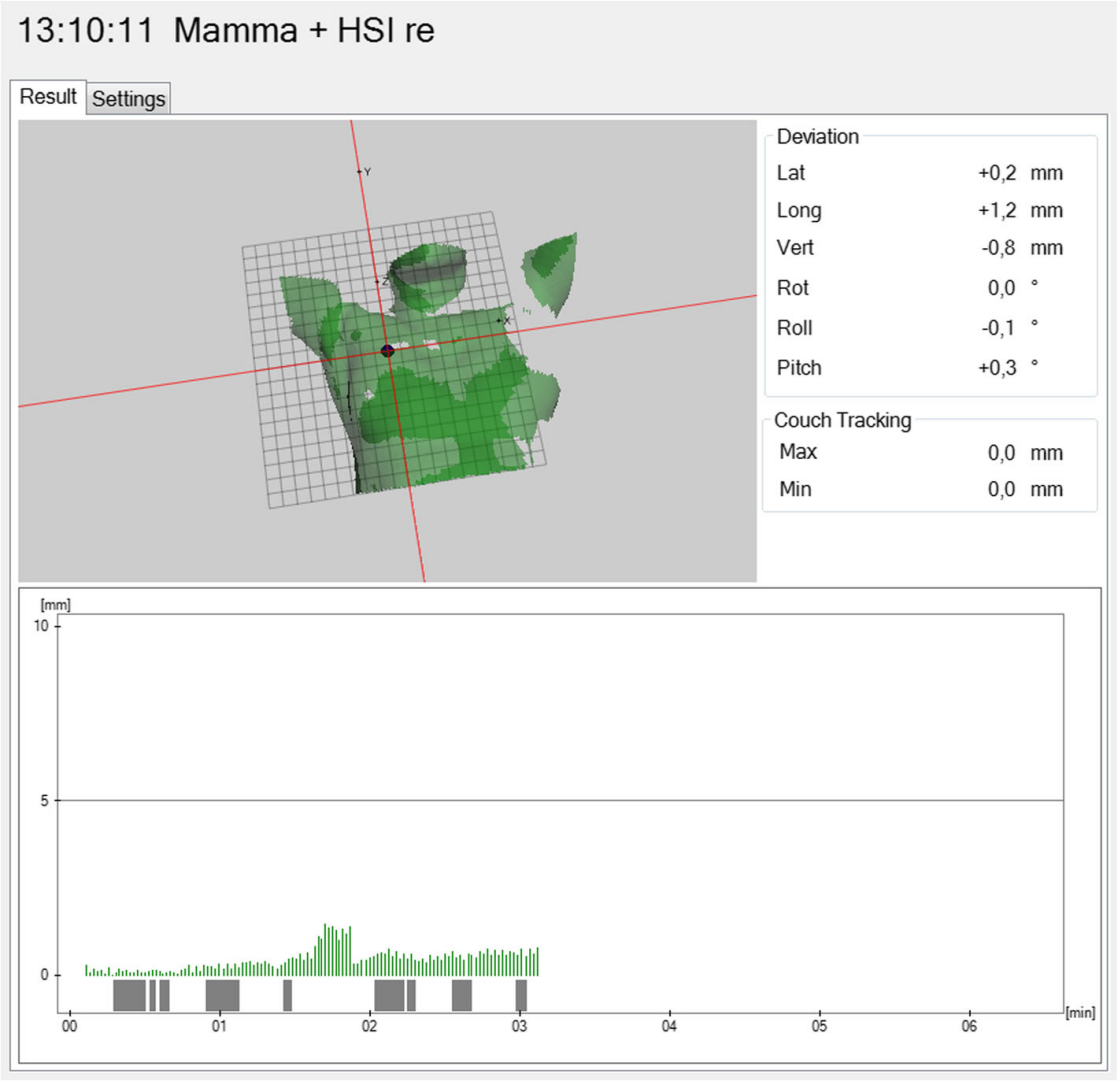
Screenshot of the c4D-software showing a patient surface image of a Catalyst scan (isocentre marked as point on the right chest wall); below the image, the measurements during a treatment session are depicted over time. Bars indicate the magnitude of the deviation vector in millimeter, and the bars above the time scale indicate the beam-status (on vs. off)

Data retrieved from the software included time stamps, cartesian coordinates including lateral, longitudinal and vertical position deviations in millimeters, the deviation/magnitude vector: $\sqrt{{\boldsymbol{x}}^{\mathbf{2}}+{\boldsymbol{y}}^{\mathbf{2}}+{\boldsymbol{z}}^{\mathbf{2}}}=\sqrt{{\boldsymbol{lateral}}^{\mathbf{2}}+{\boldsymbol{longitudinal}}^{\mathbf{2}}+{\boldsymbol{vertical}}^{\mathbf{2}}}$, rot-, roll- and pitch- angles as well as a boolean variable that shows the beam status (on vs. off). Figure 2 shows a screenshot of the c4D-tool.

All values were stored into a database and for every treatment session the corresponding data sets were exported from the c4D-software with a built-in function into a *.skv-file. To extract the data from these files we wrote a MATLAB script. All *.skv-data sets were imported into the MATLAB workspace and a consistency check was performed: whether data points were outliers or if a table shift was performed during the treatment session. In fact, all treatment sessions where portal images were acquired were not evaluated to exclude confounding. In cases of an observable table shift during an active Catalyst recording session, the treatment session had to be excluded from analysis. Additionally the motion results of every single treatment session were manually checked for plausibility in addition to an automatic outlier-measurement.

### Patient and tumour characteristics

The present study cohort included 104 patients with a median age at diagnosis of 59 years (range: 27–86 years). Tumour localization was mainly right-sided (65/104, 62.5%), as many patients presenting with left-side breast cancer were treated using a DIBH technique, which was an exclusion criterion for the present study. Most patients had early stage breast cancer, classified pT1 (50/104, 48%) or pT2 (36/104, 34.6%) with mainly negative nodal status pN0 (69/104, 66.3%). Overall, 62 patients (59.6%) received a conventional fractionated RT regimen to the breast or chest wall, 17% received an additional irradiation of the medial supraclavicular lymph nodes and 16% received a sequential boost to the tumour bed. Moreover, 42 patients (40.4%) received a hypofractionated treatment plan. 84.6% of all patients had an adjuvant radiotherapy after a breast conserving surgery, only 15.4% received a chest wall irradiation after mastectomy. Table 1 gives an overview including absolute and relative incidences for different clinicopathological and radiotherapy parameters.

**Table 1:** Descriptive patient characteristics and radiotherapy parameters of the study cohort (*n* = 104)

	No. (%)
Age at diagnosis (yrs.)	
mean ± SD	59.7 ± 13.3 years
median (range)	59.0 (50.0–70.0) years
Tumour localisation	
left	39 (37.5%)
right	65 (62.5%)
Tumour stage	
pTis	10 (9.6%)
pT1	50 (48%)
pT2	36 (34.6%)
pT3	4 (3.8%)
pT4	4 (3.8%)
Nodal status	
pN0	69 (66.3%)
pN1	22 (21.2%)
pN2	9 (8.7%)
pNx	4 (3.8%)
Fractionation	
Normo-fractionated (2/50 Gy)	62 (59.6%)
Hypo-fractionated (2.67/40 Gy)	42 (40.4%)
Radiotherapy	
Whole-Breast	88 (84.6%)
Chest-wall	16 (15.4%)

### Statistical analyses

All data from the Catalyst software c4D were analyzed. To find the maximum deviation during a treatment session, we searched for the maximum deviation of the deviation vector from the isocentre, as well as the maximum value along every single axis (lateral, longitudinal and vertical). Results were further classified into two subgroups, one group containing only measurements/samples during beam-on time and the other group including all measurements during the whole treatment session from first beam-on to last beam-off, which was defined as session time.

A t-test was used to evaluate whether the mean deviations along the three axes were statistically significant compared to zero. Additionally, for analyzing differences in the distributions between the three different possible directions (lateral, longitudinal and vertical) Friedman’s test for coupled samples and a Bonferroni method as post-hoc analysis were applied. Furthermore Mann-Whitney-U-tests were used to compare independent data samples.

Correlation analysis between different parameters was performed using Kendall rank correlation coefficient. A robust trimmed two one-sided t-test (TOST) by Yuen and Dixon (1973) was used for equivalence testing (parameters: α = 0.05, ε = 0.05, tr = 0.2). [18] As none of the parameters in this study was normally distributed, in order to reliable describe data distributions also additional information including median, quantiles and 95%-confidence intervals are given. For all statistical analyses a significance level of α = 0.05 was defined.

MATLAB Release 2016a (The MathWorks, Inc., Natick, Massachusetts, United States) was utilized for data extraction as well as data processing and R 3.3.2 with libraries ggplot2, plot3D, R.matlab, equivalence for statistical analyses.

## Results

### Descriptive analysis

Overall, 2028 treatment sessions during beam on time as well as during the whole treatment session (including the time during gantry movements) were evaluated. The mean treatment session time was 154 s (standard deviation [SD] = 53 s) (95%-confidence interval [CI]: [79–268] seconds), and the mean beam-on time was 55 ± 16 s (95%- CI: [31–90] seconds).

Table 2 shows mean, median and 95-CI of the *maximum displacements* during a treatment fraction on the one hand (*n* = 2028 sessions), furthermore split into the two subgroups. During beam-on time only the maximum of the deviation vector was 1.93 ± 1.14 mm (median = 1.63 mm) and 95%-CI [0.48–4.65] mm as compared to 2.34 ± 1.40 mm (median = 2.03 mm) and 95%-CI [0.78–5.35] mm for the whole treatment session time. Over 99% of the beam-on time, the magnitude of the deviation vector was less than 4.45 mm.

**Table 2:** Descriptive analysis of displacements along the axes divided into maximum displacement during a treatment session, and all samples of a treatment session. Furthermore, the observation results are split into samples during beam-on time only and the whole treatment session time (*n* = 104 patients, sessions = 2028)

	Beam on time only			Whole treatment session time		
*Maximum displacement per session* *(N = 2028)*	Mean ± Standard deviation [mm]	Median [mm]	95%-CI [mm]	Mean ± Standard deviation [mm]	Median [mm]	95%-CI [mm]
Lateral	0.18 ± 1.06	0.38	[−1.88–2.1]	−0.04 ± 1.39	−0.25	[−2.67–2.3]
Longitudinal	0.17 ± 1.32	0.34	[−2.89–3.11]	0.10 ± 1.71	0.45	[−3.36–3.23]
Vertical	0.68 ± 1.53	0.84	[−2.9–3.55]	0.70 ± 1.87	1.07	[− 3.62–3.74]
Magnitude of deviation-vector	1.93 ± 1.14	1.63	[0.48–4.65]	2.34 ± 1.40	2.03	[0.78–5.35]
*All samples per session*						
Lateral	0.08 ± 0.65	0.06	[−1.24–1.48]	0.07 ± 0.67	0.06	[−1.32–1.49]
Longitudinal	0.09 ± 0.81	0.04	[− 1.79–2.17]	0.10 ± 0.84	0.05	[−1.78–2.15]
Vertical	0.39 ± 0.98	0.36	[− 1.64–2.60]	0.43 ± 1.03	0.40	[−1.79–2.67]
Magnitude of deviation-vector	1.12 ± 0.98	0.78	[0.11–3.77]	1.19 ± 0.99	0.88	[0.13–3.8]

**Fig. 3:**
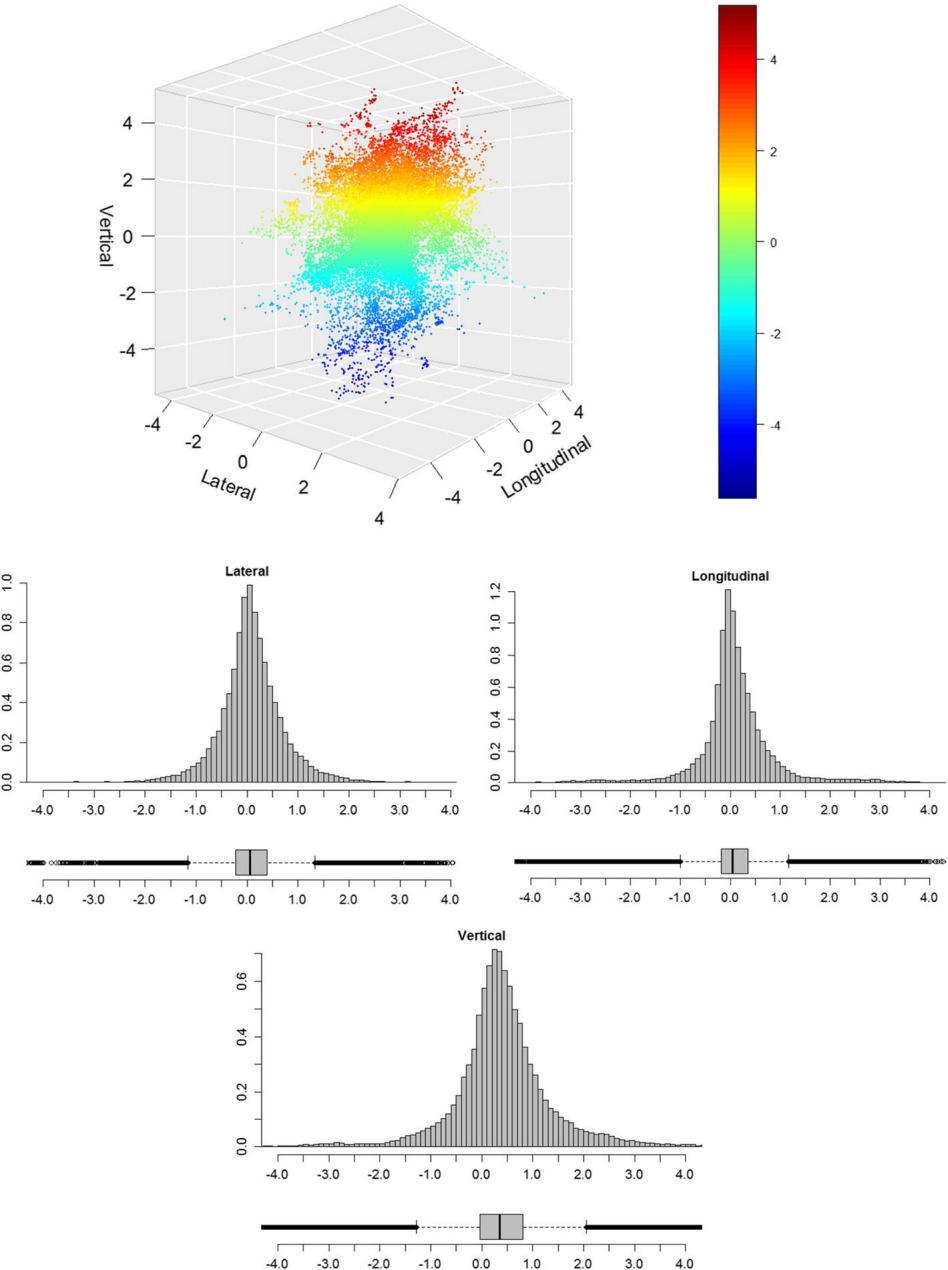
3D - scatter plot showing deviation around the isocentre including all patients and all fractions during the beam-on time (vertical deviation in color); additional histograms and boxplots for lateral, longitudinal and vertical axes (*N* = 104 patients, 69,654 points)

**Fig. 4:**
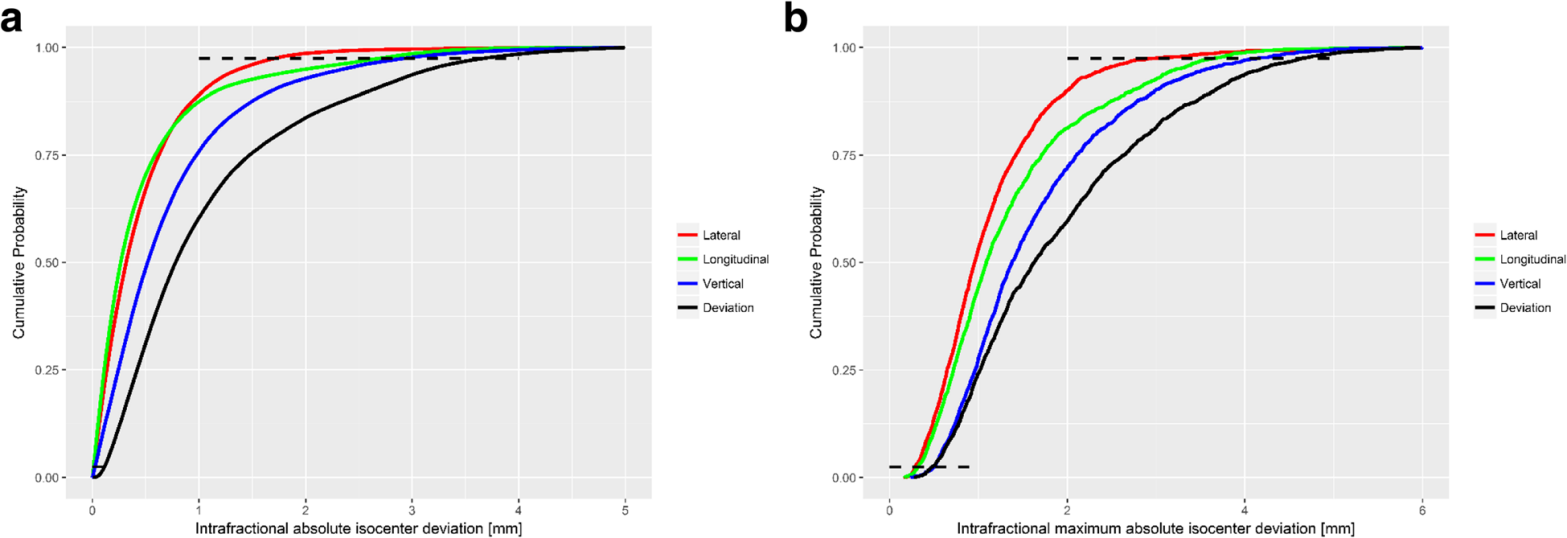
**a** empirical cumulative distribution functions for absolute isocentre deviations in millimeters along the three spatial axes and the deviation vector during beam-on-time; **b** empirical cumulative distribution functions for maximum absolute isocentre deviations; horizontal dashed black lines mark lower [0.025] and upper bound [0.975] of 95%-confidence interval

As a further analysis in the second part of Table 2, *all samples* were evaluated and reported for the two subgroups. In this case, the results between the two groups were quite similar. The magnitude of the deviation vector during beam-on time was 1.12 ± 0.98 mm, the median magnitude 0.78 mm (95%-CI: [0.11–3.77] mm). The lateral deviation was in mean = 0.08 ± 0.65 mm, median = 0.06 mm, 95%-CI: [− 1.24–1.48] mm, longitudinal mean = 0.09 ± 0.81 mm, median = 0.04 mm, 95%-CI: [− 1.79–2.17] mm and vertical mean = 0.39 ± 0.98 mm, median = 0.36 mm, 95%-CI: [− 1.64–2.60] mm. Figure 3 is a 3D-scatter plot of all samples during beam-on time (for all patients and all fractions) in lateral, longitudinal and vertical direction. It represents an overview of how the data samples are distributed in 3D-space. Additionally, the three histograms with boxplots visualize the distributions along the three axes. Figure 4 shows empirical cumulative distribution functions for absolute isocentre deviations along the three spatial axes and the deviation vector during beam-on-time.

### Analysis of distributions

For all three axes the results of t-tests analyzing the mean deviations when compared to a value of zero were statistically significant (*p* < 0.01), which means that there is a drift of the isocentre during treatment along all axes significantly larger than zero. Friedmans rank analysis for coupled samples for maximum displacement during beam-on time resulted in a significant difference of the distributions along the three axes (Chi-square(2) = 470.53, *p* < 0.01, *n* = 2028). Post-hoc Dunn-Bonferroni-Tests did not show a distribution similarity between any two of the three directions.

Mann-Whitney-U-tests for the irradiation side (left vs. right) did not show any significant difference in the maximum deviation (*p = 0.85*), also there was no difference between breast and thoracic wall irradiation (*p = 0.92*).

### Correlation analysis

Correlation analysis showed a weak correlation between maximum magnitude of deviation and time of treatment session (Kendall-Tau = 0.082, *p* < 0.01). Furthermore, between vertical and longitudinal (Kendall-Tau = 0.362, *p* < 0.01) deviation, as well as between vertical and lateral (Kendall-Tau = − 0.031, *p* = 0.04) deviation a significant correlation was verified.

### Two one sided t-test for equivalence

TOST for equivalence between beam-on time only and the whole session time of maximum displacement for all three directions was not significant (*p* > 0.05). This has to be interpreted as a dissimilarity of values, with larger variations during beam-off time, for example during gantry movement. By looking at all samples, for the lateral and longitudinal direction a similarity was verified (*p* < 0.01) between beam-on time only and the whole session time, but not for the vertical direction (*p* = 0.06).

## Discussion

The present study faces the issue of quality assurance during breast irradiation: for this aim, intra-fractional monitoring of the patients’ surface was evaluated using an optical non-invasive method. Moreover, to our knowledge, the present experience reports and discusses the data of the largest patient population (104 patients and over 2000 treatment sessions) in the setting of intra-fractional motion management by using an optical surface imaging system for breast cancer RT. Since the maximum single deviation during treatment delivery may be interpreted as a “worst case” deviation during a single treatment session and could influence adequate coverage within the applied setup margins (5 mm CTV to PTV), we have analyzed them separately from all the other samples recorded during every treatment session.

During beam-on time only a *maximum deviation* (magnitude of vector calculated as shown in Fig. 4) of mean 1.93 ± 1.14 mm (95%-CI: 0.48–4.65 mm) was found. Along the lateral axis the deviations were smaller (95%-CI: -1.88 – 2.1 mm) than on the longitudinal (95%-CI: -2.89 – 3.11 mm) or vertical (95%-CI: -2.9 – 3.55 mm) axis. Therefore, we were able to show in the present study that even this “worst case” momentary deviation was within the limits of applied setup margins, while most of the time, deviations were abundantly within the PTV margins. In fact, *all samples* during beam-on time, reported a lateral deviation of mean 0.08 ± 0.65 mm, longitudinal mean 0.09 ± 0.81 mm and vertical mean 0.39 ± 0.98 mm. The cumulative distribution functions for the spatial axes and the deviation vector (Fig. 4) gives an overview of the deviation distribution.

Various techniques for intra-fractional motion analysis in breast irradiation are reported and discussed in literature. X-ray imaging as described by Hirata et al. represents one of the options. [19] The authors used orthogonal X-ray imaging on 23 patients before and after a treatment session to observe internal target motions referring to surgical clips displacements in accelerated partial breast irradiation. The magnitude of intra-fractional motion was depending on the direction, e.g. it showed a systemic baseline drift of 1.5 mm in the posterior direction but also depending on patients’ characteristics and tumour cavity location. Yue et al. pursued a similar approach by comparing marker- and bone-based matching resulting in an average intra-fractional motion magnitude of 4.2 mm vs. 2.6 mm. [20] Later, Yue et al. described tumour side dependent directional motion along the lateral axis between left- and right-sided breast cancer patients. [21]

Other authors applied continuous portal imaging with a sample frequency of 7.5 Hz during the delivery of two tangential fields in free-breathing modality on breast cancer patients. The amplitude of the intra-fractional chest wall motion had a mean value of 2.0 ± 0.7 mm and a maximum value of 8 mm. [22] The main disadvantages of X-ray imaging in motion analysis are represented by the additional radiation exposure and the restriction to bone- or marker-based matching strategies. Especially in the case of bone-matching, results may differ from the actual tissue movement. Nevertheless, the obtained results remained within 1 cm in any direction and therefore comparable to the results of the experience reported in the present study.

Another strategy was applied by the group of Kinoshita et al., who positioned a gold marker near the nipple on the skin of breast cancer patients and used a fluoroscopic real-time tracking system for monitoring. Baseline shift and range of motion stayed within a few millimeters. [23] An optical approach method to measure intra-fractional motions was recently reported by Ricotti et al. [12], who used an optical system with infrared reflective surface markers for motion tracking. Based on their high sample frequency, the investigators were able to divide the motion into a baseline drift and respiratory-caused changes. Measurements were quite time-consuming because a calibration before every session was required and patients needed several surface markers positioned on their chest wall. In contrast, in our here reported experience, no additional markers were required. However, the findings reported by Ricotti and colleagues seem to be comparable to our analyses; the authors calculated a baseline drift of 0 ± 1.1 mm in the lateral direction, − 0.5 ± 1.7 mm in the longitudinal and − 1.4 ± 1.8 mm in the vertical direction. [12]

Regarding the influence of duration of the RT session, Wiant et al. analyzed data of 33 patients during 831 monitoring sessions. Most patients stayed within a 5 mm drift range. [24] However, the authors reported a strong correlation between patient drift and duration of treatment session. It is noteworthy that in contrast to the present study, the mean observation time was nearly twice as long (mean 342 s vs. 154 s). This underlines the fact, that treatment duration might strongly influence intra-fractional baseline drifts. Similarly, Jensen et al. used a self-developed laser-system for measuring displacements along the anterior-posterior (AP)-direction for intrafraction motion in breast cancer RT, showing a baseline drift in the posterior direction of − 1.3 mm at the end of the treatment session. Approximately 4% of treatments had a larger baseline drift than 5 mm at 5 min. [25] In our series, the mean treatment session time was very short with 154 s (95%CI: 79–268 s), thus resulting in a very weak correlation between duration of RT session and the maximum deviation of displacement.

As a more modern approach, Acharya et al. analyzed intra-fractional motion using magnetic resonance imaging (MR) guided-IMRT and calculated an intra-fractional tumour bed cavity displacement of about 0.6 ± 0.4 mm along the longitudinal and vertical axis. According to their results, the mean difference between planned and delivered dose was within 1 %. [26] Similarly, another group used MRI with two time scales (2D and 3D MRI) showing a median deviation of about 2 vs. 2.2 mm. [27] MR-imaging during RT represents an elegant and promising approach to track target volumes in real time, especially in soft tissues including breast. Nevertheless, compared to other here described optical tracking systems, these image guided systems still remain very expensive and currently not widely available in clinical practice.

One of the limitations of the present study is represented by the increased deviation vectors during gantry rotation. At certain angles, the gantry can mask one or two of the three cameras of the Catalyst system, causing misleading measurement outputs. This situation can be observed in higher deviation values during the whole treatment session time as compared to the results of the beam-on time only (see Table 2). Nevertheless, considering that only patients receiving 3D conformal RT, with a fixed gantry position during beam-on time, were recruited in the present study, this technical issue can be estimated of minor relevance for the present analysis.

In summary, intra-fraction motion during 2028 sessions of breast cancer RT observed in the present study was maintained within 5 mm in any direction as the confidence intervals show. Although specific advices on the size of PTV margins are not provided by current guidelines (since they should be based on actual measurements of set-up performance), the here reported deviations are within the clinically most common standard PTV-setup margins of 5 mm. [16] One further aspect of implications of the present findings for treatment planning could be the skin flashing of treatment fields in breast IMRT. Intensity extension with the help of auto-flash tools usually aims to account for intra-fractional patient motion or set-up errors. Based on the present results, typically used margins of up to 2.5 cm could be substantially reduced. [28]

## Conclusion

Due to the large number of data analyzed (2028 sessions in 104 patients) and the detailed reported results of specific deviations (< 5 mm), the optical real-time surface imaging system utilized in the present study showed to be an important tool for intra-fraction motion management in breast cancer RT. Not less important, the here discussed surface imaging system with visible light represents the peculiarity to be absolutely safe for patients, as it is a non-invasive approach to monitor patients position in real time without any additional radiation exposure.

## Abbreviations

CCD: Charged-coupled device
CI: Confidence interval
CTV: Clinical target volume
DIBH: Deep inspiration breath-hold
EPID: Electronic portal imaging device
IMRT: Intensity modulated radiation therapy
MRI: Magnetic resonance imaging
PTV: Planning target volume
RT: Radiotherapy
SD: Standard deviation
TOST: Two-one-sided t-tests
VMAT: Volumetric Arc Therapy
